# Cardiac Amyloidosis Presenting as Biventricular Systolic Heart Failure

**DOI:** 10.1155/2021/6671469

**Published:** 2021-04-01

**Authors:** Monique Oye, Aaron Richardson, Edin Sadic, Ahmad Alkhasawneh, Gladys Velarde

**Affiliations:** ^1^Department of Internal Medicine, UF Health Jacksonville, Jacksonville, FL, USA; ^2^Division of Cardiology, UF Health Jacksonville, Jacksonville, FL, USA; ^3^Department of Pathology, University of Florida Health Jacksonville, Jacksonville, FL, USA

## Abstract

A previously healthy octogenarian presented with new onset heart failure symptoms. Comprehensive multimodality imaging including complete echocardiography with longitudinal strain analysis, cardiac magnetic resonance imaging (cMRI), nuclear medicine pyrophosphate (99-mcTcPYP) scan along with biomarker, monoclonal protein analysis, and fat pad biopsy confirmed diagnosis of transthyretin cardiac amyloidosis.

## 1. History of Presentation

An 81-year-old female of Jamaican descent with no prior past medical history or hospitalizations presented to the hospital with complaints of four-week history of dizziness, dyspnea on exertion, and lower extremity edema. She denied angina, productive cough, and neurologic symptoms. She was hypertensive with blood pressure of 147/101 mm Hg, regular heart rate of 94 beats/minute, afebrile with 100% oxygen saturation on room air. The electrocardiogram showed sinus rhythm without acute changes. Chest radiograph revealed cardiomegaly and pulmonary congestion. Chemistry panel was unremarkable with normal renal and hepatic function, with no electrolyte abnormalities. NT-Pro-B-type natriuretic peptide was elevated at 6,000 pg/mL. Troponin T was mildly elevated at (0.07 *μ*g/L). Patient was admitted to cardiology service for further evaluation.

## 2. Investigations

**Figure 1:**
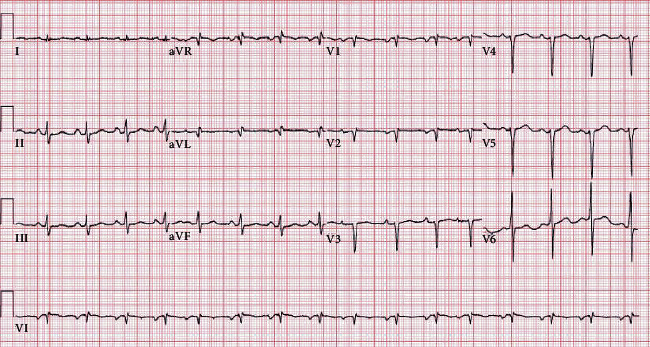
Electrocardiogram obtained on admission. Low voltage is noted in leads I, AVL; however, no left ventricular hypertrophy is noted. Q waves are seen in leads V1 and V2.

A 12-lead electrocardiogram (ECG) obtained on presentation revealed normal sinus rhythm, low QRS voltage, poor R-wave progression, and Q waves in anteroseptal leads (Figure 1). Transthoracic echocardiography (TTE) revealed a severely decreased ejection fraction (EF) of 10-15%, with biventricular systolic dysfunction, moderate mitral regurgitation, severe tricuspid regurgitation, a trace pericardial effusion, and marked biatrial enlargement. Left ventricular (LV) cavity size was noted to be small, with abnormal left ventricular mass and increased relative wall thickness of 0.78.

**Figure 2:**
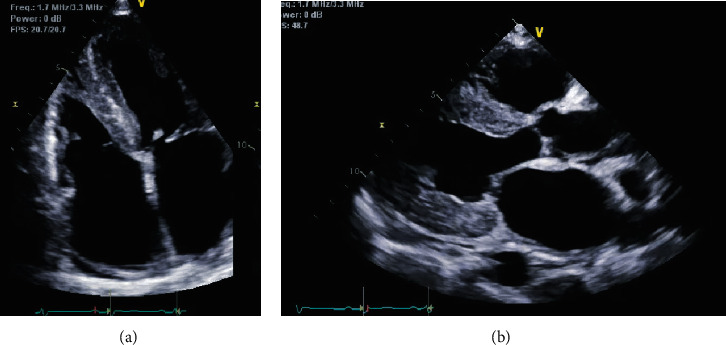
4-chamber (a) and a parasternal long axis echocardiogram view. Significant increased left ventricular wall thickness and biatrial enlargement can be seen.

**Figure 3:**
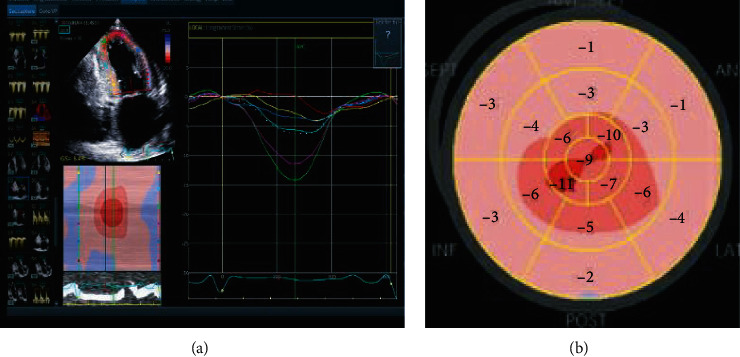
Decreased global longitudinal strain pattern on echocardiogram. There is apical sparing of longitudinal strain, a finding typically seen in cardiac amyloidosis.

Right ventricle was moderately enlarged, and right ventricular systolic function was severely impaired. The PA systolic pressure was estimated to be 40 mmHg based on complete tricuspid regurgitation spectral doppler signal. Diastolic profile was suggestive of restrictive LV filling pattern. There was abnormal global longitudinal (GL) strain with apical longitudinal strain preservation, a pattern suggestive of cardiac amyloidosis (CA) (Figures 2 and 3).

**Figure 4:**
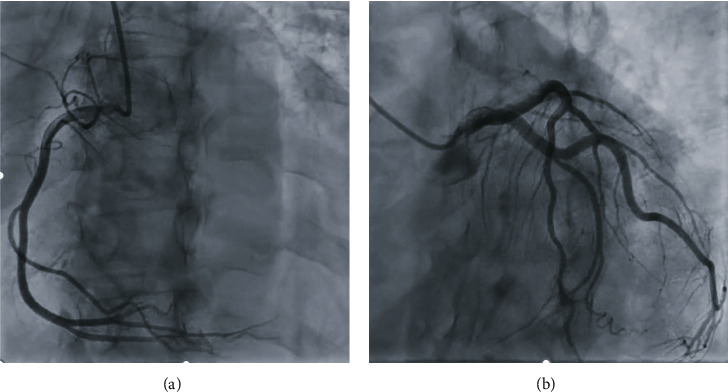
Left anterior oblique caudal images from left heart catheterization. The right coronary system (a) and left coronary system (b) appear angiographically normal.

To rule out obstructive coronary artery disease, a left heart cardiac catheterization with coronary angiography was performed. This showed angiographically normal coronaries (Figure 4) and an LVEDP of 12-14 mmHg.

**Figure 5:**
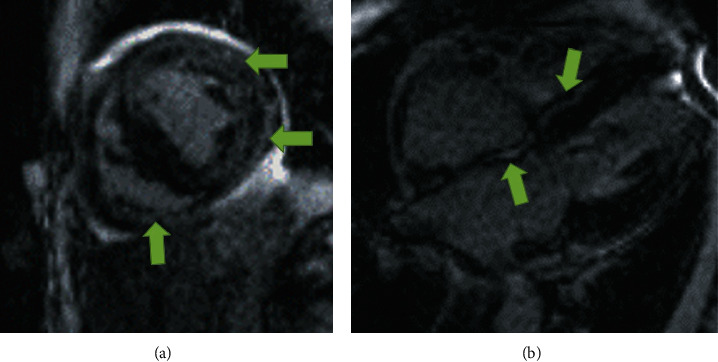
Delayed gadolinium enhancement cardiac MRI images. The image on (a) shows a short axis view, (b) is a 4-chamber view. Extensive midmyocardial and atrial wall enhancement secondary to amyloid deposition is noted (green arrows) which is a signature of cardiac amyloidosis.

Cardiac MRI was then pursued, which revealed areas of subendocardial delayed gadolinium enhancement within the left ventricle not conforming to a vascular territory. This was also noted throughout the left atrium and along the septal subendocardial surface of the right ventricle (Figure 5). These features favored a diagnosis of infiltrative cardiomyopathy. Laboratory markers were ordered to assess for evidence of a monoclonal protein. Serum immunofixation electrophoresis (IFE) revealed a monoclonal spike (0.38 g/dL) in the gamma region corresponding to an IgG ambda paraprotein. Urine IFE had no evidence of monoclonal paraprotein. Kappa/Lambda free light chain ratio was normal at 1.58 mg/L, which made AL subtype less likely.

**Figure 6:**
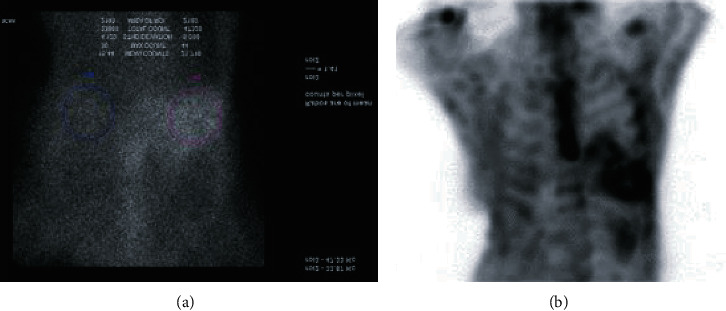
Pyrophosphate scan (99mTc-PYP). Increased cardiac uptake can be seen in the image in (a) denoted by purple circle.

**Figure 7:**
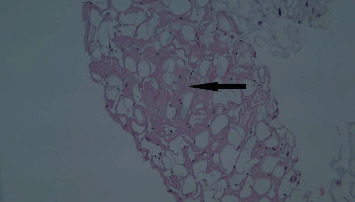
Amyloidosis in abdominal fat. Abdominal fat pad biopsy revealed pretentious infiltrate (arrow) which shows apple-green birefringence on red stain, consistent with amyloidosis (H&E stain, 100x).

In the setting of monoclonal gammopathy of undetermined significance (MGUS) a nuclear medicine pyrophosphate scan (99mTc-PYP) was performed to distinguish between light chain (AL) amyloidosis and transthyretin (TTR) amyloidosis. 99mTc-PYP scan showed grade 2/3 myocardial radiotracer uptake suggestive of TTR amyloidosis (Figure 6). The heart to contralateral (H/CL) ratio was 1.41. Fat pad aspirate biopsy showed positive staining for Congo red (Figure 7). Bone marrow biopsy did not reveal overt evidence of involvement by plasma cell neoplasm. Genetic testing did not reveal any mutations of the TTR gene. Patient was ultimately diagnosed with wild-type transthyretin amyloidosis (wt-ATTR).

## 3. Management

The patient presented in New York Heart Association (NYHA) functional Class III. Her heart failure symptoms were managed primarily with intravenous loop diuretics to achieve euvolemia. An attempt was made to start standard guideline directed therapy with beta-blockers and angiotensin converting enzyme inhibitors (ACEI), when diagnosis was in question, but she was unable to tolerate them due to hypotension.

With the use of diuretics, her functional status markedly improved, and she was able to return to performing her usual daily activities. Her symptoms improved to NYHA Class II.

## 4. Discussion

Amyloidosis is a commonly underdiagnosed cause of cardiomyopathy [1]. The two most common subtypes that affect the heart include light chain (AL) amyloidosis and transthyretin (ATTR) amyloidosis [1]. AL is caused by extracellular deposition of misfolded immunoglobulin light chains secreted by clonal plasma cells in the bone marrow [2]. AL cardiac amyloidosis (CA) is a rare condition that can affect nearly every organ in the body, leading to multiorgan failure. The clinical course of AL CA is more virulent and accelerated. Treatment for AL subtype typically includes chemotherapy ± autologous stem cell transplant.

ATTR results from deposition of misfolded TTR, a protein tetramer that is produced in the liver and deposits as insoluble amyloid in extracellular spaces causing damage to the heart and or peripheral nerves. ATTR CA can be wild-type (ATTR-wt, age-related) or variant (ATTRv, hereditary) [1]. ATTR-wt-CA, previously known as senile systemic amyloidosis, is usually diagnosed at an older age, has a male predominance, and is caused by age-related misfolding of genetically unaltered TTR.

ATTRv-CA is an autosomal-dominant disease in which gene mutations lead to changes in the protein TTR [3]. The presentation of hereditary ATTRv-CA depends on the type of mutation present. Some cause familial amyloid polyneuropathy (FAP), others familial amyloid cardiomyopathy (FAC), and some FAP/FAC overlap syndromes. It is usually diagnosed at younger ages, and signs of neuropathy (i.e., carpal tunnel syndrome) typically precede cardiomyopathy by years in advance [1]. In the United States, transthyretin isoleucine 122 (Val122Ile) is a common variant of hereditary ATTRv-CA, and this genetic mutation disproportionally affects African American patients [4]. This variant results in a late-onset, restrictive cardiomyopathy that can mimic other forms of heart failure, such as hypertensive heart disease, leading to underdiagnosis [4].

To avoid misdiagnosis, genetic testing for the Val122Ile mutation can play a role in identification and treatment for patients as well as their family members. Novel treatments are being developed for amyloidosis (ATTR) subtypes that can slow the progression of cardiomyopathy by working as transthyretin silencers and stabilizers [1].

Clinically significant cardiac involvement in both types of TTR typically presents as a syndrome of heart failure with preserved ejection fraction [2], abnormal troponin levels, and markedly elevated pro-bnp levels. However, heart failure with reduced ejection fraction can be seen in the advanced stages of the disease. The gold standard for diagnosis is endomyocardial biopsy. However, recent advances in imaging have provided noninvasive modalities that are helpful in identifying cardiac amyloid. Characteristic patterns on electrocardiogram, echocardiogram, and cardiac MRI can assist in earlier detection. Red flag features for cardiac amyloid typically seen on echocardiogram include increase in left ventricular (LV) wall thickness (relative wall thickness > 0.6), small LV chamber size, speckled myocardium, atrial enlargement, and diastolic dysfunction with a restrictive diastolic pattern [1]. Additionally, relative apical sparing of longitudinal strain (LS) is considered a specific finding [5]. Previous studies have demonstrated that relative sparing of LS in the LV apex using 2-D speckle tracking is highly sensitive (93%) and specific (82%) for the diagnosis of cardiac amyloid [5].

Cardiac MRI is another useful imaging modality showing various patterns of late gadolinium enhancement. However, it is unable to distinguish between the AL and TTR subtype [6].

Nuclear imaging technique with bone-seeking radiotracers have become a diagnostic method for ATTR-CA and is able to distinguish it from AL-CA with high specificity [7]. Different radiotracers can be used including technetium-3,3-diphosphono-1,2-propanodicarboxylic acid (^99m^Tc-DPD) and ^99m^Tc-PYP. ^99m^Tc-DPD scintigraphy is currently the most studied isotope in ATTR-CA and has widespread use among amyloid centers in Europe [8]. It is not approved for clinical use in the United States, where ^99m^Tc-PYP scanning is more commonly utilized. There has yet to be a direct comparison of the different radiotracers to determine if one has greater specificity in diagnosing ATTR-CA [8]. The exact mechanism by which these bone-seeking radiotracers accumulate in the myocardium of patients with cardiac amyloidosis remains unclear; however, studies suggest an association between radiotracer myocardial uptake and amyloid fibril type in patients with ATTRv [9].

Our patient was an elderly African American female, presenting de novo with significantly increased ventricular wall thickness and heart failure of uncertain etiology. While keeping more prevalent pathologies in the differential; it is important to consider amyloidosis as a potential etiology in this demographic despite her age, sex, and race. While it is true that ATTR-wt CA appears to be more common in older white men, it has also been shown to be present in up to 50% of elderly women admitted with heart failure in at least one prospective study where 99mTc-PYP imaging was used with high accuracy of detection of ATTR-wt cases [10]. Similarly, studies evaluating ATTR-wt have almost exclusively consisted of Caucasian patients. However, population-based data from the United Kingdom have reported an equal prevalence of ATTR-wt among races [9]. Underrecognition of this disease may explain why African Americans are poorly represented in American studies of ATTR wt-CA [11].

Regardless of patient's age, TTR genetic testing should be performed in all patients with ATTR-CA since the results have significant implications for family members at risk as mentioned earlier.

Our case illustrates the occurrence of advanced biventricular systolic heart failure in an elderly woman with ATTR-CA. Her course had been rather indolent, but this condition can be in fact undiagnosed or underdiagnosed for years as many “subtle” signs can be attributed to the aging process.

Her case is unique as this condition typically present with heart failure with preserved ejection fraction (HFpEF). The right ventricle (RV) is usually normal or thickened, especially in the anterior wall, and if the RV is involved, signs of pulmonary hypertension may be observed. In the advanced phases of the disease, biventricular systolic function deteriorates. This has substantial clinical implications, as treatment strategies may become even more limited.

Usual guideline directed medical therapy with angiotensin-converting enzyme inhibitors (ACE-I), and beta-blockers for heart failure with reduced EF (HFreF) have not been shown to offer clinical benefit. In fact, most medications have potential to cause harm as they may exacerbate hypotension when amyloid-associated autonomic dysfunction is present or further limits cardiac output due to relatively fixed stroke volumes secondary to restrictive filling [7]. Accurate diagnosis of ATTR-CA is important to avoid the potentially deleterious effects of using these medications in this group of patients. Furthermore, the benefits of implantable cardioverter defibrillator (ICDs), particularly for primary prevention of sudden cardiac death in patients with HFreF and ATTR-CA, have not been well established. ICDs are recommended only in cases of aborted sudden cardiac death with expected survival >1 year or significant ventricular arrhythmias [7] Our patient did not have ventricular arrhythmias, and ICD placement was deferred. She was referred to the heart failure clinic on discharge for outpatient follow-up and potential treatment with novel ATTR therapies.

## 5. Conclusions

This case highlights the different imaging modalities that can be used to diagnose cardiac amyloidosis. Echocardiogram and electrocardiogram findings in conjunction with cardiac MRI can assist with the initial diagnosis when amyloidosis is suspected. Nuclear imaging with 99mTc-PYP can further distinguish between different types of amyloidosis (AL vs. ATTR).

Although endomyocardial biopsy is the gold standard diagnostic test, hematology testing, fat pad, genetic testing, and bone marrow biopsies can aid in confirming the diagnosis without putting patients at risk for complications with endomyocardial biopsy. Early detection can lead to an increased number of patients being referred for treatment.

## Additional Points

*Take Home Messages/Learning Objectives*. (i) Recognize the different imaging modalities used in the diagnosis of cardiac amyloidosis. (ii) Recognize how to distinguish the different subtypes of cardiac amyloidosis. (iii) Recognize the common echocardiogram findings seen in cardiac amyloidosis.
